# Homeostatic Responses to Subsystolic Arterial Occlusive Pressure in Glabrous and Non-Glabrous Skin Circulation

**DOI:** 10.3390/biomedicines14040888

**Published:** 2026-04-13

**Authors:** Joana Caetano, Pedro de la Villa Polo, José Delgado Alves, Luis Monteiro Rodrigues

**Affiliations:** 1 Systemic Autoimmune Diseases Unit, Department of Medicine IV, Fernando Fonseca Hospital, 2720-276 Amadora, Portugal; joana.r.caetano@ulsasi.min-saude.pt (J.C.); 2 CBIOS—Research Center for Biosciences & Health Technologies, Universidade Lusófona, Campo Grande 376, 1749-024 Lisboa, Portugal; 3 Systems Biology Unit and Escuela Doctorado, Facultad de Medicina y Ciencias de la Salud, Universidad de Alcalá, 28871 Alcalá de Henares, Madrid, Spain; pedro.villa@uah.es; 4 Immune Response and Vascular Disease, iNOVA4Health, Nova Medical School, Faculdade de Ciências Médicas, Universidade Nova de Lisboa, 1169-056 Lisboa, Portugal; jose.alves@nms.unl.pt (J.D.A.)

**Keywords:** reactive hyperemia, sub-occlusive arterial compression, glabrous and non-glabrous skin, sympathetic control

## Abstract

**Background:** Reactive hyperemia (RH) is used to assess microcirculatory function in vivo and has traditionally been interpreted as a local, ischemia-driven vasodilatory response following arterial occlusion. However, perfusion changes consistently observed in contralateral, non-challenged limbs question the exclusively local nature of RH. **Objective:** This study aimed to characterize reactive hyperemic responses elicited by subsystolic cuff pressures, below arterial occlusion pressure (AOP), and to investigate their effects on glabrous and non-glabrous skin microcirculation and on global hemodynamics. **Methods:** Seven healthy women underwent a standardized protocol consisting of baseline stabilization, a 2 min subsystolic cuff inflation (70–80% of resting AOP) in one arm, and a recovery period. Microvascular perfusion was simultaneously assessed in both hands using laser Doppler flowmetry (LDF) on glabrous skin and polarized light spectroscopy (PSp) on non-glabrous dorsal skin. Hemodynamic indicators were continuously monitored using CNAP (Continuous Non-invasive Arterial Pressure) technology. Ipsilateral and contralateral responses were compared across experimental phases. **Results:** Subsystolic cuff inflation induced significant perfusion changes not only in the challenged limb but also in the contralateral limb, despite the absence of a complete arterial occlusion. **Conclusions:** These findings confirm the adaptive nature of RH emphasizing the major role for the sympathetic nervous system in glabrous skin. In glabrous (palmar) skin, a similar perfusion profile is shown in both hands but significant differences could only be found in the ipsilateral hand. In contrast, non-glabrous (dorsal) skin demonstrated region-specific increases in perfusion, again evident in the ipsilateral hand, suggesting venous stasis. No changes in global hemodynamic variables were observed throughout the protocol. Further studies in larger, more diverse populations are needed to confirm these observations and refine the mechanistic understanding of reactive hyperemia.

## 1. Introduction

Reactive hyperemia (RH) is a well-known method to evaluate microcirculatory function in vivo [1,2,3,4]. It is often defined as an increase in blood flow within a given vascular territory induced by controlled occlusion of a major artery, commonly the brachial or femoral artery [5,6,7]. The post-occlusive reactive hyperemia (PORH) maneuver requires a pressure cuff that, once inflated with suprasystolic pressure, reduces the downstream (blood) flow, while the subsequent deflation evokes a fast reperfusion and vasodilation. This response is reportedly linked to local ischemia-related vasodilators and myogenic responses, and detectable reductions in related endpoints have been associated with impaired microvascular function in the measured region [6,7,8,9,10]. However, the nature of the adaptive mechanisms involved with RH is still equivocal, limiting the clear understanding of the involved mechanisms and the clinical applicability of the method [4,5,6,11].

Our investigations in the upper and lower limbs have shown that when a stressor is applied to one limb, a measurable response appears simultaneously in the contralateral limb [12,13,14,15]. It is the case of a brief, low-intensity massage applied to one lower limb, which induces a significant, reproducible perfusion increase in the contralateral limb [12]. Likewise, the classical venoarteriolar reflex (VAR) changes perfusion in the resting contralateral limb during the maneuver and after it ceases [13]. Hyperemic responses similar to PORH can be elicited by any challenge that alters local perfusion conditions, even without major-vessel involvement [12,14]. These findings, which cannot be related to ischemia or to local adaptations, parallel hyperemic responses observed after application of PORH to the upper limb. Furthermore, our group recently demonstrated the presence of similar adaptive responses in the upper and lower limbs resulting from a classical PORH applied in one randomly chosen arm [16].

In the present study, we further examine these hyperemic responses obtained in the absence of a complete arterial occlusion, with a pressure below the arterial occlusion pressure (AOP), the minimum cuff pressure required to completely stop arterial blood flow into a limb [17,18,19]. Therefore, this exploratory study is designed to determine if the same fast-occurring neuronal reflex, already visible during occlusion and after the cuff release [15,16], is present with subsystolic pressure during this partially occlusive procedure, and to determine how this impacts skin circulation in glabrous and non-glabrous skin and global hemodynamics. We believe this research will help to dissociate ischemia and reactive hyperemia and add more mechanistic information regarding the cardiocirculatory adaptive responses involved in RH.

## 2. Materials and Methods

Seven healthy women (mean age 34.1 ± 8.2 years old) were selected (Table 1) following general inclusion–non-inclusion criteria common to this type of study [20]. Participants were instructed not to consume caffeine or alcohol or engage in intensive exercise up to 24 h before the experiments. Measurements were obtained by the same investigator in a room with controlled temperature (22 ± 1 °C) and humidity (40–60%). All procedures complied with the Declaration of Helsinki and its subsequent amendments [21] and were previously approved by the Lusofona University’s Health Sciences Ethics Committee (process CE.ECTS/P10.21) in September 2021.

During measurements, participants were seated comfortably with both forearms resting on a surface near chest-height, both hands facing palm down. The AOP was determined by gradually inflating a cuff on the arm until arterial pulse signals were not detectable at the radial artery (confirmed by laser Doppler flowmetry). The pressure was set within a 70–80% range of the resting AOP, that is, approximately 30 mmHg below the participant’s systolic pressure. The procedure consisted of phase 1—stabilization (10 min), phase 2—challenge with a subsystolic cuff pressure maintained for 2 min, and phase 3—recovery after cuff deflation (10 min). The contralateral limb was used as a control.

Peripheral circulatory changes were detected in both hands by two non-invasive optical technologies—laser Doppler flowmetry (LDF) [22,23] and polarized light spectroscopy (PSp) [24,25,26]. Although both rely on the interaction of light and skin, these technologies measure different variables. Table 2 summarizes the main differences between technologies, which are further discussed ahead.

LDF probes were placed on the ventral side of the second finger of each hand, enabling continuous perfusion acquisition (40 Hz) expressed in arbitrary blood perfusion units (BPUs) (MoorVMS-LDF, Moor Instruments, Axminster, Devon, UK). In the dorsal skin of both hands, microcirculation was measured without contact using the PSp system (TiVi701 Tissue Viability Image System, WheelsBridge, Linköping, Sweden) and expressed as the concentration of red blood cells (CRBC, in arbitrary units). A coupled digital camera placed 30–60 cm above the table surface, provided real-time video images of microcirculation obtained using cross-polarization filters in each of three selected regions of interest (ROI). Three ROIs were “1” the middle region between the proximal interphalangeal and distal interphalangeal of the fourth finger, “2” the region immediately below the fourth metacarpophalangeal, and “3” the middle region of the wrist internally to the cubital styloid apophysis.

Advanced hemodynamics (mean arterial pressure, peripheral vascular resistance and systolic volume) was monitored using a Continuous Non-invasive Arterial Pressure (CNAP) system (CNAP^®^ technology, CNSystems Medizintechnik GmbH, Graz, Austria) [27,28]. Measuring cuffs were applied in the second and third fingers in the contralateral (non-occluded) limb.

Data was analyzed using Prism 8 software (GraphPad Software, LLC, Boston, MA, USA). Normality was assessed using the Shapiro–Wilk test. Mean values of LDF skin perfusion and temperature, skin perfusion at each ROI, and hemodynamic variables were analyzed in each phase. The Wilcoxon matched pairs signed-rank test was used for phase comparison of hemodynamic measures within each group. The Friedman test was used for analysis of three or more repeated measures within each group. A post hoc analysis (Dunn’s test) was performed when the Friedman test showed significant statistical results (*p* < 0.05). A confidence interval of 95% and a *p* < 0.05 was considered for statistical significance.

## 3. Results

**Figure 1:**
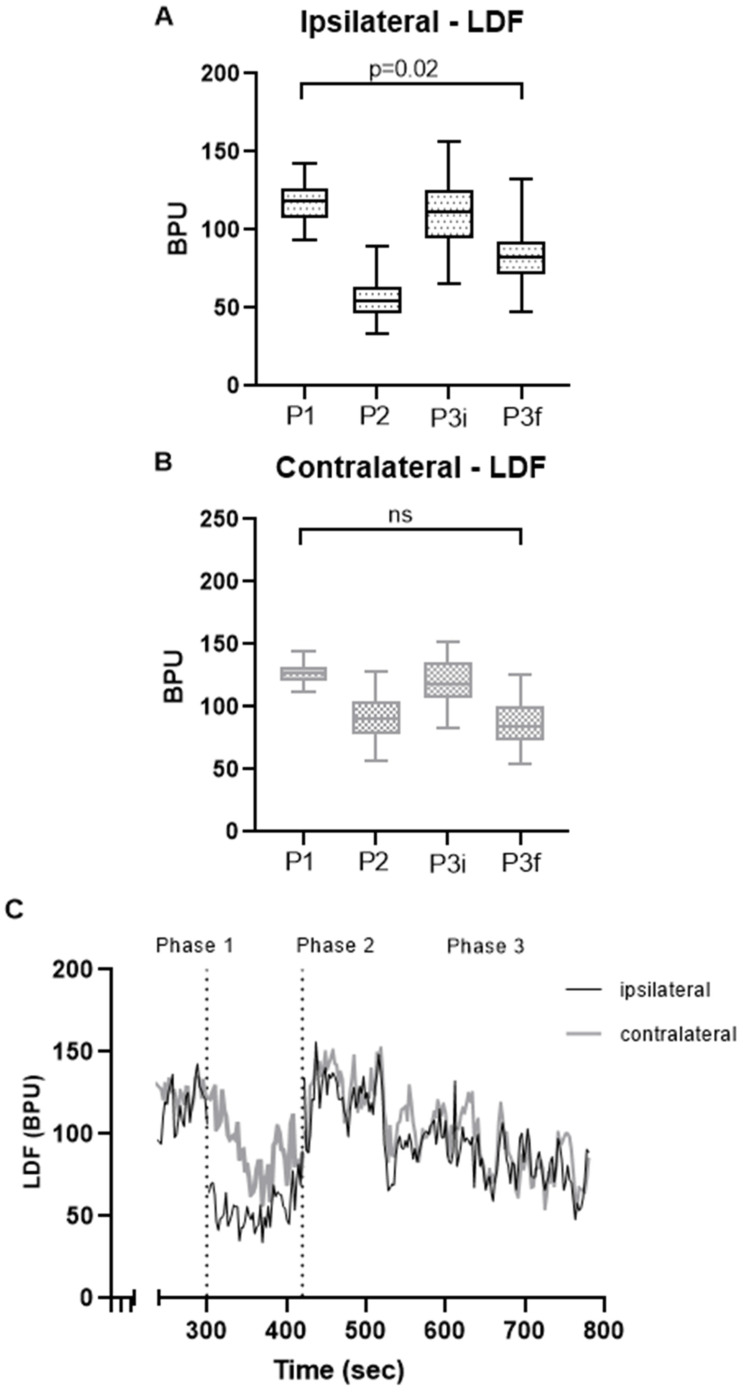
Perfusion changes detected during the protocol in the ventral (palmar) face of both hands with LDF. Box plots (**A**,**B**) represent median values and interquartile ranges. (**C**) represents mean perfusion (all participants) register during the experiment. Phase 1 data represents stabilized perfusion in the last minute before occlusion. Friedman test was used for comparison, followed by a post hoc analysis with Dunn’s test in ipsilateral arm, showing a significant difference in perfusion between phase 2 and 3i (*p* = 0.01) in the ipsilateral hand. P1—phase 1; P2—phase 2; P3—phase 3; P3i—first minute of phase 3 (initial recovery); P3f—last 3 min of phase 3 for final recovery; BPU—blood perfusion units; ns—non-significant.

**Figure 2:**
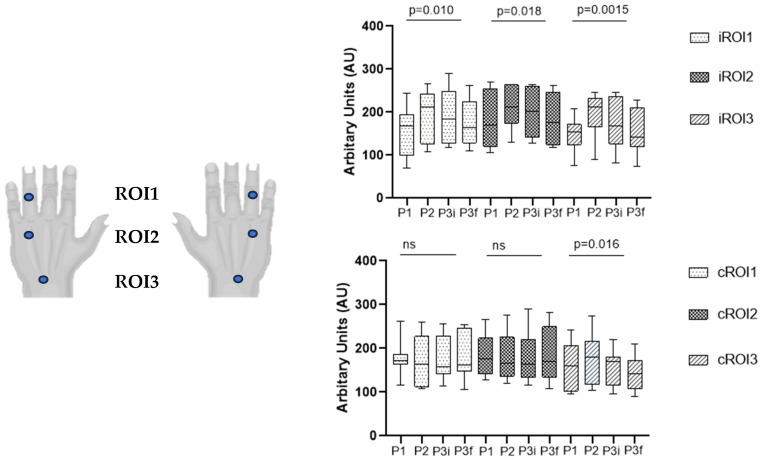
Perfusion changes detected with PSp TiVi in the dorsal skin of both hands in each region of interest—ROI (i—in the ipsilateral hand; c—in the contralateral). Box plots represent median values and interquartile ranges. Freidman test was used for comparison, followed by a post hoc analysis with Dunn’s test, showing a significant difference in ipsilateral arm measurements: ROI1—between P1 and P2 (*p* = 0.023), ROI2—between P2 and P3f (*p* = 0.023), ROI3—between P2 and P3f (*p* = 0.006); in contralateral arm ROI3—between P2 and P3f (*p* = 0.011). (P1—last minute of phase 1; P2—phase 2; P3—phase 3; P3i—first minute of phase 3 (initial recovery); P3f—last 3 min of phase 3 for final recovery; BPU—blood perfusion units; ns—non-significant.

Responses obtained with subsystolic pressure (within the 70–80% range of resting AOP) and simultaneously registered in glabrous and non-glabrous skin of both hands are shown in Figure 1 and Figure 2 respectively. Regarding the glabrous palmar skin, LDF profiles were similar in both hands. As shown (Figure 1) the pressure increase in the arm evoked a clear decrease in perfusion in the ipsilateral limb. A similar, though milder, response was also registered in the contralateral (non-challenged) hand. Significant differences were noted in the ipsilateral limb between baseline (phase 2) and recovery (phase 3).

The current investigation also involved the dorsal skin of both hands, which is structurally different from palmar skin, with a denser epidermis and a deeper vascular plexus adapted to its physiological functions. These two types of skin reflect different physiological (thermal, mechanical, sensorial) functions, and both have been reported to have a strong sympathetic control that, in dorsal skin, also involves a vasodilator component [29]. We recorded data from three regions of interest on the dorsal skin of both hands following the fourth finger, along the longitudinal axis, from the wrist to the fingertip. Microcirculation here assessed with the contactless PSp TiVi system showed significant increases in CRBC at the finger and in the wrist ROIs in the ipsilateral hand (Figure 2). In the contralateral hand, CRBC differences could only be found in the wrist ROI. Global hemodynamics did not change during the procedure (Table 3).

## 4. Discussion

RH has been described as a local phenomenon, consisting of an intense vasodilation evoked by a combination of flow modification and ischemia [5,6,10,30,31]. In the present study, we assessed the adaptive microcirculatory responses to subsystolic arterial occlusive pressure using two optical-based technologies—LDF and PSp—to register the effects of the intervention in both glabrous and non-glabrous skin, simultaneously. These two technologies, although related, differ in important ways (Table 2). Several studies have been published to compare the behavior of PSp (TiVi) to reference technologies such as LDF in multiple experimental contexts, including RH. All studies agree that both technologies can detect vasodilation, despite differences in spatial and temporal resolution. Moreover, strong correlations between perfusion and CRBC can be observed when the experimental design appropriately accounts for the methodological specificities of each technique [32,33,34].

Our observations have consistently pointed to the homeostatic adaptive nature of these responses. In particular, we emphasize the key role of the neural component of this response, visible with occlusion (phase 2), which evokes a consistent vasoconstriction that is present in the contralateral non-challenged limb until occlusion ceases [20]. Furthermore, global hemodynamics did not change during the procedure (Table 3), again corroborating the homeostatic nature of the response confined to the affected area. Multiple factors (dimension, circulatory architecture, autoregulation) have been implied to explain this nature [35].

The involvement of the nervous system in RH and its autonomic component are not novelties [36,37,38,39]. However, the response’s trigger is still under debate. Recently, our group documented the sequence of real-time events registered during PORH with laser Doppler flowmetry in the fingertips (glabrous) and with photoacoustic imaging (PAT) in the forearm (non-glabrous) skin, confirming a sequence of intense constriction and vasodilation occurring simultaneously in both territories [20]. As we noted, the sudden reduction in pressure and flow in the downstream vessels following occlusion should be linked to the autonomic reflex that elicits the transient hypoperfusion, hypoxia, and acidosis. Not surprisingly, occlusion is recognized as an important determinant in the PORH maneuver, dependent on the cuff pressure to elicit the post-occlusive adaptive response. Recent data confirmed that a nonlinear relationship between AOP and blood flow exists between 40 and 80% of the resting (r)AOP. No changes were noted in pressures higher than 130% rAOP [40,41,42]. We have previously shown that the RH response can also be elicited in the absence of occlusion of a major vessel, with the response proportional to the challenge intensity, in line with recent observations on muscular passive distension [43,44]. Cuff pressures lower than AOP have been used to obtain venous occlusion in vivo [45,46]. However, its confirmation with the available measurement technologies is still challenging.

The complexities of human skin circulatory control must be considered [47,48]. The cutaneous circulation is not merely a passive vascular bed but a highly specialized, reflex-regulated network that plays a critical role in thermoregulation and hemodynamics. Structural and functional differences between glabrous and non-glabrous skin are particularly relevant in interpreting perfusion responses to restrictive challenges as in the present case.

In glabrous skin arteriovenous anastomoses (AVAs) are the dominant vascular structures. These specialized shunt vessels allow blood to bypass capillary networks and are richly innervated by sympathetic adrenergic fibers, particularly those expressing α2-adrenergic receptors [49,50,51]. Their dense sympathetic control makes this region especially responsive to changes in central sympathetic outflow. Under resting conditions, sympathetic tone maintains a partial constriction of AVAs. During our challenge, the reduction in arterial inflow and perfusion pressure further decreases microvascular perfusion, producing the characteristic drop observed in phase 2 in the ipsilateral hand. Although the contralateral hand demonstrates a broadly similar perfusion profile, no statistically significant differences were identified (Figure 1). When the occlusion challenge ceases, perfusion rapidly increases, marking the early recovery phase. This immediate reperfusion is primarily attributable to withdrawal of sympathetic vasoconstrictor tone (sympathetic withdrawal), resulting in transient vasodilation of as a consequence of a lower resistance. The abrupt reopening of previously constricted pathways permits a short-lived hyperemic response. Following this initial surge, a slower and more sustained recovery phase (late recovery, phase 3; Figure 1) ensues. Despite progressive normalization, perfusion remains significantly lower than baseline during this interval. This prolonged phase likely reflects local endothelial and microvascular regulatory mechanisms reestablishing homeostasis. Endothelium-dependent mediators—such as nitric oxide, prostacyclin, and endothelium-derived hyperpolarizing factors—gradually restore vascular tone and capillary recruitment. Additionally, metabolic by-products accumulated during the occlusion period and myogenic adjustments of resistance vessels may contribute to the delayed normalization [3,5,6,10,35,38].

Thus, the perfusion profile here observed following this partial occlusion seems to represent the integrated effect of centrally mediated sympathetic modulation, structural characteristics of AVA-rich glabrous skin, and local endothelial regulatory processes.

Non-glabrous (hairy) skin is commonly controlled by the sympathetic nervous system, showing arterioles as the main resistance vessel [51,52]. Results show that our procedure induces a sequence of events suggesting the occlusion evokes venous stasis (Figure 2). Differences are more visible in the ipsilateral hand, visible in ROI1, ROI2, and ROI3, but only in ROI3 in the contralateral hand. Compared to palmar skin, this region is less sensitive to shunting, allowing direct capillary–venular pooling, meaning that venous congestion might be the most predictable and proportional consequence of cuff pressure under these experimental conditions. However, these pressures have also shown evidence of higher sympathetic activation [19,53,54] which recalls previous references to a sympathetic cholinergic vasodilator system linked to the regulation of body temperature and hemodynamics in non-glabrous skin. Mechanisms driving this activity remain unclear, potentially involving an unknown neurotransmitter acting alongside acetylcholine from sympathetic cholinergic fibers [47,53].

In conclusion, this data clearly underscores the adaptive character of RH and emphasizes the major role of the sympathetic nervous system in the adaptive response, especially in glabrous skin, aligning with our previous publications.

There are obvious limitations related to the limited sample size and the study’s exploratory character. However, we note that all rAOPs were determined for each patient and set between 70% and 80% range of relevant rAOP to ensure the referred pressure–occlusion relationship. It is worth noting that both LDF and PSp technologies were used, specifically considering the respective specificities previously identified (Table 2).

Nevertheless, these data will need to be further explored in larger and more diverse populations to increase the robustness of our findings.

## Figures and Tables

**Table 1:** Global characterization of participants enrolled in the study. Results represent mean and standard deviation.

Age (years old)	34.1 ± 8.2
BMI, kg/m^2^	21.4 ± 2.5
Body Mass (kg)	57.4 ± 6.0
Height (m)	1.64 ± 0.07
Mean Arterial Pressure (mmHg)	83.2 ± 7.8
SYS (mmHg)	115.3 ± 6.5
DIA (mmHg)	63.2 ± 4.7
Ankle–brachial index (ABI)	1.05 ± 0.07
Physical Activity (h/week)	4.7 ± 2.3

BMI—body mass index; h/week—number of hours per week; m—meter.

**Table 2:** Summary of the technical specifications of the laser Doppler flowmetry (LDF) and Tissue Viability Imaging (TiVi).

	LDF	TiVi
Biophysical principle	Optical with contact/Doppler effect	Optical non-contact/Polarization spectroscopy
Light frequency	~780 nm	~500 nm
Sampling	Single point measurement	Wider Region of Interest (ROI)
Measurement Area/Depth/Tissue	~1 mm^2^/~0.5 mm/at the papillary and reticular dermis	900–1600 cm^2^/~0.5 mm/at the papillary and reticular dermis
Physiological variable/units	Perfusion/arbitrary Blood Perfusion Units (BPUs)	Concentration of Red Blood Cells—CRBC/AUs
Spatial resolution	n.a.	50 μm
Temporal resolution	30–40 Hz	Up to 25 images/s

n.a. not applicable.

**Table 3:** Hemodynamics recorded in the contralateral arm during experimental procedures. Comparative statistics (Wilcoxon) between phases are also shown.

	Phase 1	Phase 2	Phase 3	*p* Phase 1 vs. 2	*p* Phase 1 vs. 3	*p* Phase 2 vs. 3
Systolic Arterial Pressure (mmHg)	111.10 ± 6.74	119.9 ± 9.52	116.8 ± 9.56	0.156	0.375	0.546
Mean Arterial Pressure (mmHg)	80.75 ± 5.57	85.56 ± 10.24	84.61 ± 6.66	0.297	0.156	0.578
Diastolic Arterial Pressure (mmHg)	61.40 ± 6.40	65.91 ± 7.64	66.43 ± 4.19	0.469	0.047	0.297
Heart Rate (bpm)	76.62 ± 8.31	75.34 ± 7.75	77.09 ± 9.41	0.078	0.812	0.218
Peripheral vascular resistance (dyn·s/cm^5^)	1185.00 ± 115.80	1304.00 ± 298.1	1303.00 ± 182.7	0.375	0.078	0.578
Systolic volume (mL)	66.09 ± 8.81	65.91 ± 12.42	63.56 ± 11.41	0.937	0.156	0.094

## Data Availability

The original contributions presented in this study are included in the article. Further inquiries can be directed to the corresponding author.
